# The severity of fatigue and its interplay with biological and psychological factors in people with hand osteoarthritis – Results from the Nor-Hand study

**DOI:** 10.1016/j.ocarto.2026.100747

**Published:** 2026-01-17

**Authors:** Daniel H. Bordvik, Marthe Gløersen, Elisabeth Mulrooney, Pernille Steen Pettersen, Lene Maria Sundbakk, Tuhina Neogi, Ingvild Kjeken, Ida K. Haugen

**Affiliations:** a Center for Treatment of Rheumatic and Musculoskeletal Diseases (REMEDY), Diakonhjemmet Hospital, Oslo, Norway; b Oslo Metropolitan University, OsloMET, Faculty of Health Sciences, Institute of Rehabilitation Sciences and Health Technologies, Oslo, Norway; c Rehabilitation West and the Norwegian Women's Public Health Association Haugesund, Haugesund, Norway; d Section of Rheumatology, Boston University Chobanian & Avedisian School of Medicine, USA; e Faculty of Medicine, University of Oslo, Norway

**Keywords:** Hand osteoarthritis, Fatigue, Prevalence, Network analysis

## Abstract

**Background:**

Fatigue represents a concurrent yet uncommonly addressed symptom among people with hand osteoarthritis (OA). We aimed to explore fatigue severity and its interplay with other variables among hand OA patients.

**Methods:**

We used baseline data collected in the Nor-Hand study, a cohort comprising participants with hand OA recruited at Diakonhjemmet Hospital. We measured fatigue severity by using a numeric rating scale (NRS, range: 0–10). Drawing on correlations from our dataset, existing literature, and input from a patient research partner, we estimated regularized networks to explore the interplay of fatigue. Network nodes represented variables (i.e., measures of fatigue, all bodily pain, anxiety/depression, self-efficacy, age, comorbidities, body mass index and sleep), and edges represented weighted conditional non-directional relationships between nodes. In sensitivity models, radiographic OA severity, high-sensitivity C-reactive protein, grip strength and indices of central pain sensitization were added. Network accuracy and stability were tested by bootstrapping (n = 1000).

**Results:**

Our sample included 300 participants (mean (standard deviation) age; 60.8 (6.2) years, 88 % women). The mean (SD) severity of fatigue was 4.1 (2.9) NRS points. Network analyses identified comorbidities, all bodily pain, symptoms of anxiety/depression, and sleep problems as central and strongly related to fatigue, independent of additional variables included in sensitivity analyses. Edge weights and node centrality estimates were considered accurate and stable based on bootstrapped confidence intervals.

**Conclusion:**

Fatigue is likely significant and strongly related to comorbidities, pain, symptoms of anxiety/depression, and sleep problems among hand OA patients. Longitudinal studies using more diverse samples and comprehensive variable selections are warranted.

## 1 Background

Fatigue represents a concurrent yet less commonly addressed symptom among people with hand osteoarthritis (OA) [1,2]. Fatigue, or tiredness comprising weariness or heaviness, may hamper abilities to perform tasks, work, socialize, and self-manage disease [1,3]. Fatigue prevalence in hand OA is underexplored, but patients report their severity as slightly lower than those with rheumatoid arthritis (RA) and more than twice that of age-matched healthy controls [2]. While fatigue may be modifiable, its underlying mechanisms, particularly in hand OA, remain poorly researched [4,5]. In knee and hip OA, most studies report strong associations between fatigue severity and measures of pain, sleep problems, psychological and cognitive factors, body mass index (BMI), comorbidities, disability, age, and female sex, while limited evidence also suggests an association between fatigue and levels of inflammation or radiographic changes [3,4]. Although often studied individually, these factors likely interact to sustain or worsen fatigue and may also be directly or indirectly influenced by fatigue [5]. Network models, with nodes representing variables and edges reflecting weighted conditional relationships, are ideal for examining the complex interplay of previously studied factors and identifying the most central variables beyond traditional regression models [6]. Using secondary data from a hand OA cohort, we aimed to explore the severity of fatigue and its interplay and central relationships through network estimation.

## 2 Material and methods

### 2.1 Study design and population

We used data from the baseline visit (2016–2017) of the Nor-Hand study cohort, a hospital-based observational study primarily conducted to explore OA pain at Diakonhjemmet Hospital. Participants aged 40–70 years with OA in at least one thumb base or finger joint by ultrasound or clinical examination were recruited [7]. We excluded people with systemic inflammatory joint disease or psoriasis. A flowchart is included as Supplementary Fig. 1. A rheumatologist assessed the fulfilment of the American College of Rheumatology criteria for hand OA [7]. During the Nor-Hand study planning, a patient research partner advised on data collection, including fatigue measures. The study was approved by the Norwegian Regional Committee for Medical and Health Research Ethics (Ref.no: 2014/2057) and is registered at https://clinicaltrials.gov (NCT03083548).

### 2.2 Data collection

The selection of variables was guided by available literature, patient experiences (obtained in a face-to-face meeting with our patient research partner), and correlations with fatigue in our dataset. Using a numeric rating scale (NRS, range: 0–10), participants rated their severity of fatigue based on the question “*During the last 24 h, did you experience a feeling of tiredness?*“, with the anchors “*0 = No problems with tiredness*” and “*10 = Severe problems with tiredness*”. Age and sex were obtained from medical records. We used validated questionnaires on sleep problems (five levels), fatigue self-efficacy (range: 10–100), symptoms of anxiety/depression (range: 0–42), a comorbidity index (range: 0–45) and all bodily pain (NRS, range: 0–10) [7]. BMI was estimated based on measured weight and height (kg/m^2^). Bilateral posteroanterior radiographs were obtained and scored at joint-level for OA severity in the distal and proximal interphalangeal, metacarpophalangeal, and the first carpometacarpal and interphalangeal joints and scaphotrapeziotrapezoidal joints (n = 32 joints) using the modified Kellgren-Lawrence (KL) scale (score: 0–4) and summarized to generate a radiographic OA severity (ROA) sum score (range: 0–128) [8]. Grip strength, i.e., the highest value in kilograms from three attempts, was measured by a Jamar dynamometer. The value from the dominant hand was used in analysis as a measure of function. Blood samples were analysed for levels of high-sensitivity C-reactive protein to represent levels of inflammation [9]. Mechanical temporal summation (TS) and pressure pain threshold (PPT) were measured to represent indices of central sensitization. Measurements of TS included touching a weighted punctuate probe ten times (frequency: 1 Hz) at the left distal radioulnar joint. We used the difference between the participant's NRS pain at the first touch and the peak of the fifth or tenth touch [7]. PPT was tested by using a FPIX25 Wagner digital handheld algometer, applying a steadily increasing pressure (0.5 kg/s) perpendicular to the musculus tibialis anterior until the participant reported slight pain. We used the average pressure (kg/cm^2^) from three consecutive tests [7]. The intra-class coefficient (two-way mixed effects model, absolute agreement, individual measure) of TS and PPT were 0.56 and 0.43, respectively [9]. For demographic descriptions, self-reported year of hand symptom debut and highest level of education (seven levels) were also collected [7].

### 2.3 Statistical analysis

Although no cut-off specifically addressing the patient acceptable symptom state (PASS) for fatigue among patients with hand OA exists, 4 NRS points have previously been suggested for pain in hand OA, and fatigue in RA [10,11]. Hence, we used this threshold to explore the proportion of patients reporting significant fatigue (i.e., >4 NRS-points).

We explored the interplay of fatigue in two distinct models. Our main model combined data on age, all bodily pain, sleep problems, comorbidities, symptoms of anxiety/depression, BMI, fatigue self-efficacy and fatigue [[3], [4], [5],^12^]. Considering the overrepresentation of women in our data and a low correlation (r = 0.10) between sex and fatigue, sex was excluded from the network analyses. In a sensitivity model, we added measures of ROA severity, hs-CRP, pain sensitization, and grip strength to the main model, given the underexplored or unclear relationships between fatigue and these variables [[3], [4], [5],^12^,^13^]. We assessed variable distributions for normality after which high-sensitivity C-reactive protein was log-transformed due to skewness. Measures of TS and PPT were sex-standardized, reflecting the number of standard deviations (SD) away from the sex-specific mean. We presented the model and demographic data by means, medians or proportions as appropriate.

Of the included patients, only 3 % had incomplete data on at least 1 model variable. Assuming data were missing completely at random, we imputed missing values using multiple imputation by chained equations as implemented in the R package *mice* to increase power [14]. We created 20 imputed datasets, with a maximum of 10 iterations, and compared dataset variabilities across model variables (i.e., variances). As methods like Rubins rules to estimate a pooled network accounting for between-dataset variability is not available for network analyses, we estimated and compared networks for each individual imputed dataset. We presented results from the first dataset. Additionally, we conducted a complete case analysis to compare against results from the imputed data.

All network analyses were performed with R (version 4.4.3; R Foundation for Statistical Computing (https://www.r-project.org/)). Both networks and their subsequent accuracies and centrality indices were explored using the adjoining “*R*”- packages *bootnet* and *qgraph*, as recommended [6]. We estimated parameters using the least absolute shrinkage and selection operator, a regularization technique that applies a tuning parameter (tuning = 0.25) to minimize the Extended Bayesian Information Criterion and penalize complex networks potentially with a higher number of spurious edges [6]. Confidence intervals indicating edge weight accuracies were derived by bootstrapping (n = 1000). Correlations between centrality indices from the original network and sample subsets were tested using nonparametric bootstrapped case-dropping (n = 1000). In addition to visual inspections of centrality, difference tests of bootstrapped confidence intervals implemented in *bootnet* were used [6].

## 3 Results

Demographic and clinical characteristics from the main dataset, and the non-imputed and imputed datasets, are shown in Table 1 and Supplementary Table 1, respectively. The sample mean (standard deviation) severity of fatigue was 4.1 (2.9) NRS-points, with n = 134 participants (44.7 %) reporting a value above 4 NRS points (data not shown).

**Table 1:** Sample characteristics (N = 300).

Characteristics	
Age (years), mean (SD)	60.8 (6.2)
Sex, n women (%)	266 (88.7)
Symptom duration (years), median (IQR)^a^	6 (3–13)
Education (University level), n (%)^a^	174 (58 %)
NRS fatigue (range: 0–10), mean (SD)^a^	4.1 (2.9)
Sleep problems (15D, range: 1–5), mean (SD)	2.3 (1.0)
Fatigue self-efficacy (range: 10–100), mean (SD)^a^	61.4 (22.0)
Anxiety/depression (HADS, range: 0–42), mean (SD)^a^	7.3 (6.2)
Comorbidities (range: 0–45), mean (SD)	7.7 (4.3)
NRS all bodily pain (range: 0–10), mean (SD)^a^	4.0 (2.3)
BMI (kg/m^2^), mean (SD)	26.5 (5.0)
ACR criteria for hand OA, n (%)	278 (92.7)
KL sum-score (range: 0–128), mean (SD)	29.9 (19.0)
Grip strength (kg, dominant hand), mean (SD)^a^	21.9 (9.3)
hsCRP, median (IQR)∗	1.6 (0.8–4.3)
Temporal summation (range: 0–10), mean (SD)^a^, ^b^	1.6 (1.6)
Pressure pain threshold (kg/cm^2^), mean (SD)^a^^,^^b^	5.5 (2.6)

*Abbreviations*: SD = standard deviation; IQR = inter-quartile range; HADSv= Hospital Anxiety and Depression Score; NRS = Numeric Rating Scale; BMI = Body Mass Index; kg = kilograms; m = meter; ACR = American College of Rheumatology; KL = Kellgren-Lawrence; hsCRP = high-sensitive C-Reactive Protein; cm = centimeter; IQR = inter-quartile range.

a = *missing values imputed*: Symptom duration: n = 22; Education: N = 1; NRS fatigue: N = 4; Sleep problems: N = 1; Fatigue self-efficacy: N = 3; HADS, N = 9, NRS all bodily pain: N = 3; Grip strength: N = 2; hsCRP: N = 9; Temporal summation: N = 2; Pressure pain threshold: N = 9.

b = raw values are presented, but sex standardized were used in analyses.

**Fig. 1:**
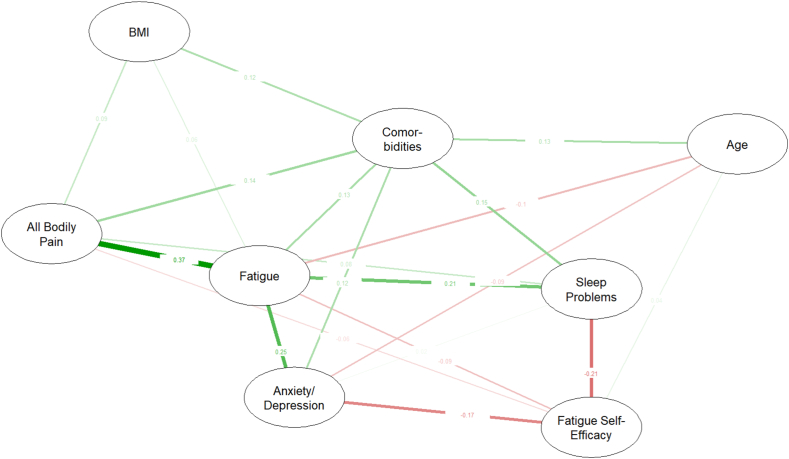
Network of fatigue in people with hand OA (N = 300). The green and red colours symbolize conditional positive and negative relationships, respectively, while thicker denser coloured edges represent conditionally higher strength. Numbers represent the weights of these regularized and partial correlations between nodes. *Abbreviations*: BMI = Body Mass Index

Fig. 1 presents the estimated network from our main model. Based on visual network inspections, and strength-plots and difference tests accessible in Supplementary Figs. 2 and 3, respectively, the most central nodes identified in addition to fatigue were comorbidities, all bodily pain, anxiety/depression and sleep problems. These results largely remained despite dropping 59 % of the sample as defined by bootstrapping, indicating good stability (Supplementary Fig. 4). The most significant edges were fatigue-pain, fatigue-anxiety/depression and fatigue-sleep problems, with accuracies (i.e., width of bootstrapped confidence intervals) mostly considered as moderate to high (Supplementary Figs. 5–6). These results were largely conserved in the sensitivity model, with the additional variables being more peripheral with no additional significant direct associations to fatigue (Supplementary Figs. 7–10). Although the relative network strength of BMI and age increased, this was largely due to a relationship with inflammation and ROA severity, respectively. Results from the presented model did not differ from the complete case analysis, or networks using the other imputed datasets (data not shown).

## 4 Discussion

To our knowledge, this is the first study to suggest proportions reporting significant fatigue in hand OA, and to explore fatigue networks in this population.

The mean NRS fatigue score in our study was 4.1 (SD 2.9). This severity numerically compares to that reported in other studies involving people with OA, and various inflammatory rheumatic diseases, and considered to be clinically relevant [2,4,15]. Using 4 NRS points as PASS, we also found that 44.7 % of participants reported fatigue above this threshold [10,11]. In contrast, previous studies report significant fatigue only in 35–41 % of OA populations, potentially due to different measurement tools [3]. However, without an established hand OA PASS for NRS fatigue, our results, which exceed previous observations, require cautious interpretation.

We also identified that comorbidities, all bodily pain, symptoms of anxiety/depression, and sleep problems were central and strongly directly or indirectly connected to fatigue. These findings align with patient-reported experiences and prior reviews based on conventional univariate or multivariate regression analyses in populations with knee OA, hip OA or RA [1,3,5,12]. While the influence of central sensitization indices on fatigue was weak, the moderate reliability of the TS and PPT measures calls for cautious interpretation. Nevertheless, the stronger relationships observed between fatigue and hand OA *illness* (e.g., all bodily pain) compared to more objective measures of hand OA *disease* (e.g., systemic inflammation and radiographic changes) in our sensitivity analyses, support prior knowledge and highlight the subjective and likely fluctuating nature of fatigue [3,5]. Notably, grip strength, a measure that could represent both hand and overall function, showed weak relationships with fatigue, contrasting previous studies [12,13]. However, these studies primarily reported on self-reported function, while the associations with performance-based function have been minimally or inconsistently assessed [12]. Also, grip strength may be more dependent on hand pain, mobility, and OA severity in our population, hampering the ability to represent overall function [13].

However, while edges may resemble mediation analyses in structure (i.e., direct and indirect effects), our networks indeed represent statistical, non-causal (i.e., bidirectional) dependencies conditional of the variables included and are best suited for generating hypotheses [6]. For example, increasing fatigue self-efficacy (e.g., through patient education) could potentially reduce fatigue directly, or indirectly by improving sleep problems and/or anxiety/depression. Previous studies support a tailored, holistic approach to fatigue [1,5].

Clinically, our networks may suggest treatment alternatives for fatigue, as the factors identified as strongly related to fatigue in our models appear modifiable, with potential overlaps in recommended non-pharmacological treatments [5]. For example, physical activity and exercise, activity pacing or cognitive behavioral therapy may improve comorbidities, pain, sleep problems, anxiety/depression, or fatigue symptoms, in people with RA or knee or hip OA [4,5]. However, these interventions, calling for an inter-disciplinary management, are underexplored on people with hand OA.

Several additional study limitations need attention. Our results may not be generalizable to men or people attending primary care. Despite its comprehensive data collection, the Nor-Hand study was not primarily designed to explore fatigue and lacked healthy controls for comparison. Although widely used, NRS fatigue may not capture the full complexity of fatigue, and “tiredness” may be confused with “sleepiness” [1,4]. Given the unestablished PASS for hand OA fatigue NRS our reported “significant fatigue” proportion may be incorrect. Despite the accuracy and stability of the models presented, our cross-sectional design, and potentially incomplete variable inclusion, especially regarding sex and optimal measures of function, prevent us from drawing causal relationships among variables [6].

In conclusion, fatigue may be significant in people with hand OA and strongly related to self-reported comorbidities, all bodily pain, anxiety/depression and sleep problems. Longitudinal studies with more diverse samples and comprehensive variable selections are needed to investigate fatigue as an outcome in hand OA.

## Author contributions

Substantial contributions to the conception and design of the study: DHB, MG, EM, TN, IK and IKH, or acquisition/analysis of data: DHB, MG, EM, PSP, LMS, and IKH. Interpretation of data and drafting of the manuscript or revising it critically: DHB, MG, EM, PSP, LMS, TN, IK and IKH. Final approval of the publication: DHB, MG, EM, PSP, LMS, TN, IK and IKH. Agreement to be accountable for all aspects of the work in ensuring that questions related to the accuracy or integrity of any part of the work are appropriately investigated and resolved: DHB, MG, EM, PSP, LMS, TN, IK and IKH.

## Funding

The Nor-Hand study was funded by the Norwegian Research Council (project number: 328657), ADVANCE grant from Pfizer/Lily, South-East Norway Regional Health Authority, Pahle's foundation, Simon Fougner Hartmann's Family foundation and Trygve Gythfeldt's research foundation. TN was supported by NIH K24 AR070892, P30 AR072571 and R01 AG066010. DHB's work was completely funded by his grant from the Norwegian Women's Public Health Association's department at Haugesund Rheumatological Hospital, Haugesund, Norway, to support his PhD-program. The funders were not involved in the study design, collection, analysis or interpretation of the current data.

## Declaration of competing interests

For transparency, IKH receives consulting fees (honoraria for lectures) from Novartis, GSK, Grünenthal and Abbvie. TN reports on consulting fees from Novartis outside the current work. LMS, MG, EM, PSP and IK have nothing to disclose.
