# Supplementary material for: The severity of fatigue and its interplay with biological and psychological factors in people with hand osteoarthritis – Results from the Nor-Hand study
Source: Osteoarthr Cartil Open. 2026 Jan 17;8(1):100747. doi: 10.1016/j.ocarto.2026.100747 (PMC12861025; doi:10.1016/j.ocarto.2026.100747)
Supplement: Multimedia component 1 [file mmc1.docx]

**Supplementary Figure 1:** Flowchart showing the process of recruitment in the Nor-Hand study, 2016-2017.
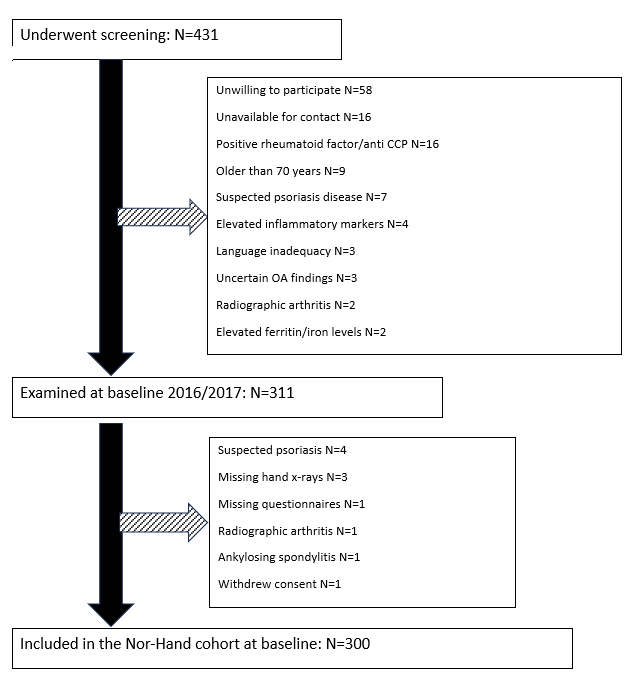


**Supplementary Figure 2:** Strength plots of the estimated network of fatigue, presented as relative values (i.e., higher values reflect higher strength). Node *strength*, the preferred metric in exploratory analyses, quantifies how well each node is directly connected to other nodes; *betweenness* quantifies how important a node is in the average path between two other nodes; *closeness* quantifies how well a node is indirectly connected to other nodes [6].


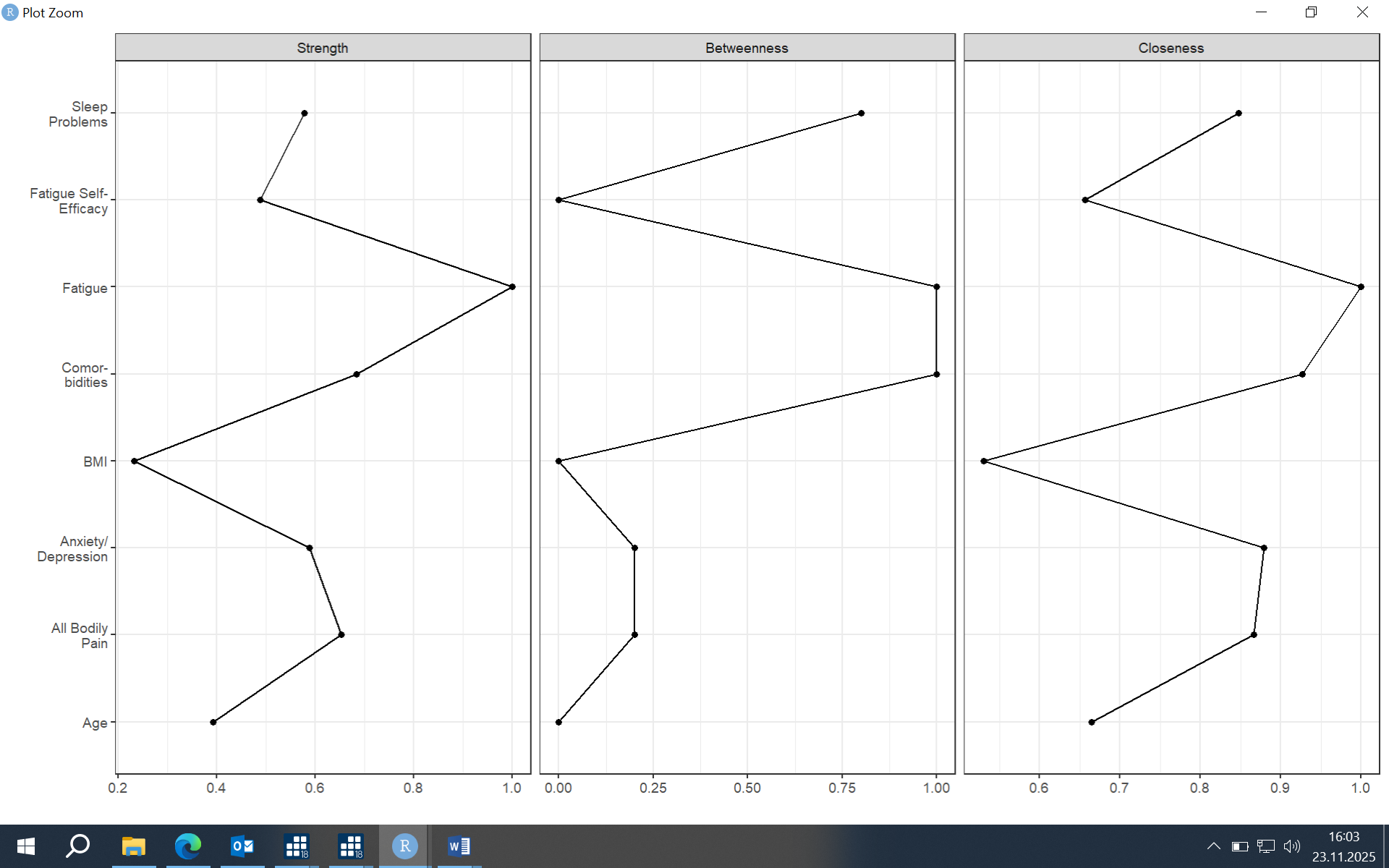


**Supplementary Figure 3:** Test of significant differences across the centrality of each node in the fatigue network in people with hand OA (N=300), estimated using bootstrapped values. Higher value represents higher centrality, while black and grey boxes represent *significantly different from* and *non-significantly different* from, respectively.


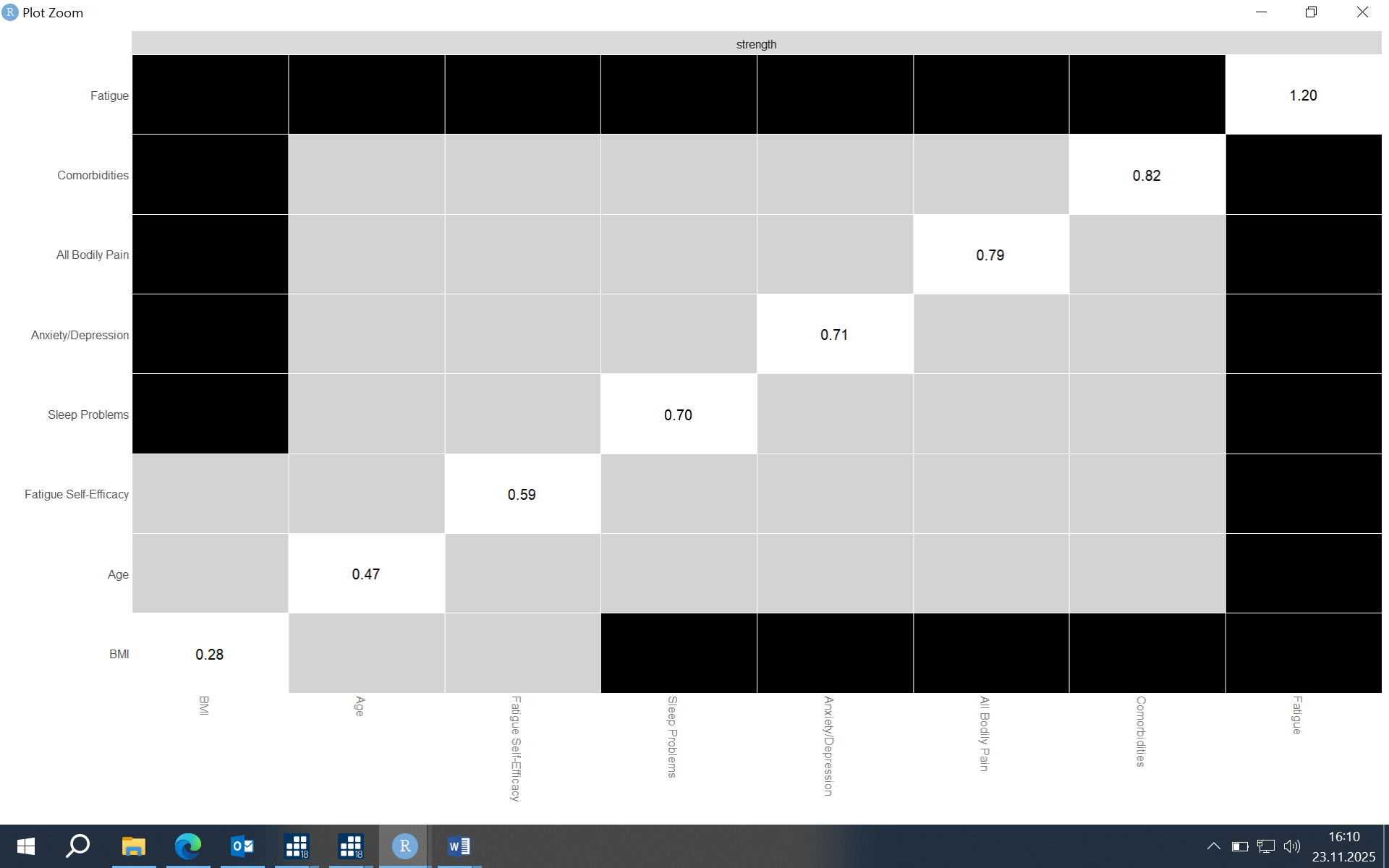


**Supplementary Figure 4:** Average correlations between centrality indices (strength) of fatigue networks comparing the original sample and samples where persons were dropped by bootstrapping (replications; n=1000) in people with hand OA (N=300). The line indicates the respective means, while areas indicate the range from the 2.5th quantile to the 97.5th quantile. In our study, the post-estimated correlation stability scores, i.e., the proportion of participants that can be dropped to still keep a correlation of 0.7 between the bootstrapped and the original node strength was 59.3%, while the same value for edges was 59%, both indicating that the order of centrality indices demonstrated good stability [6].


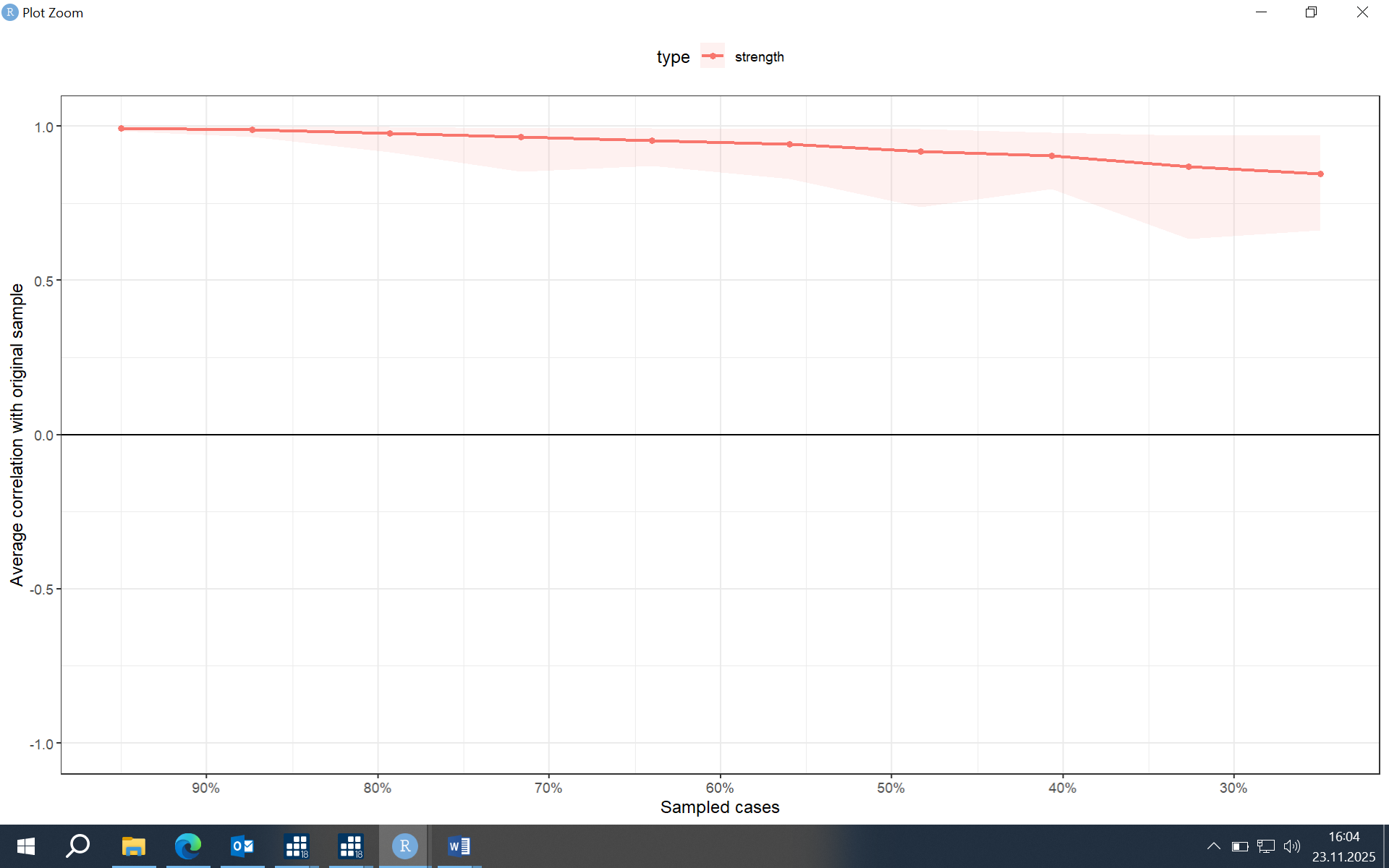


**Supplementary Figure 5:** Pooled original means and bootstrapped confidence intervals and means (replications; n=1000) for the visual assessment of edge weight accuracies in the network of fatigue in people with hand OA (N=300). *Abbreviation*: BMI = Body Mass Index


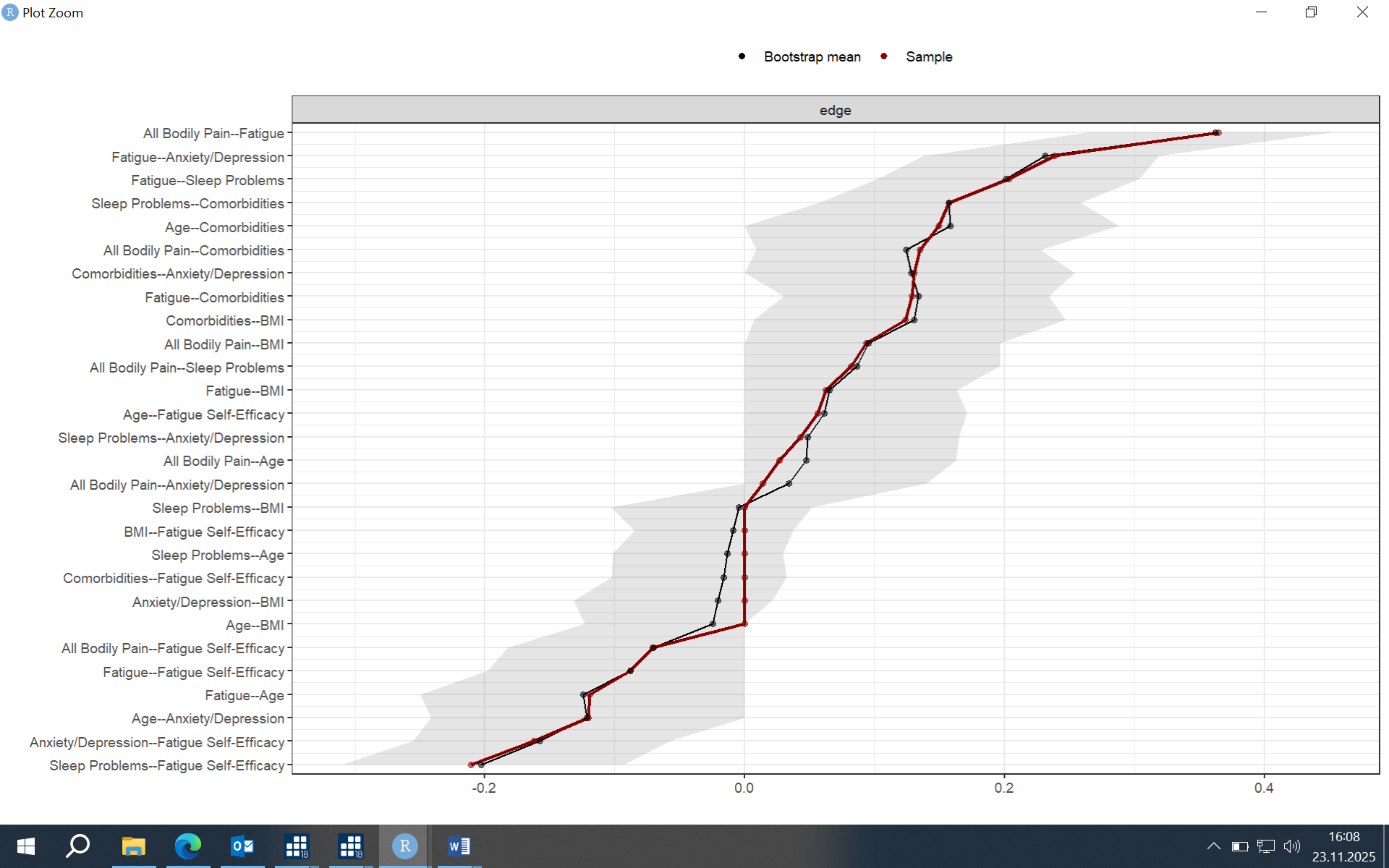


**Supplementary Figure 6:** Test of significantly different edge weights in the network of fatigue in people with hand OA (N=300), estimated as differences across bootstrapped confidence intervals. Red colours represent negative correlations, while blue colours represent positive correlations. Denser colours represent higher edge weights, while black and grey boxes represent *significantly different from* and *non-significantly different* from, respectively. Abbreviation: BMI = Body Mass Index


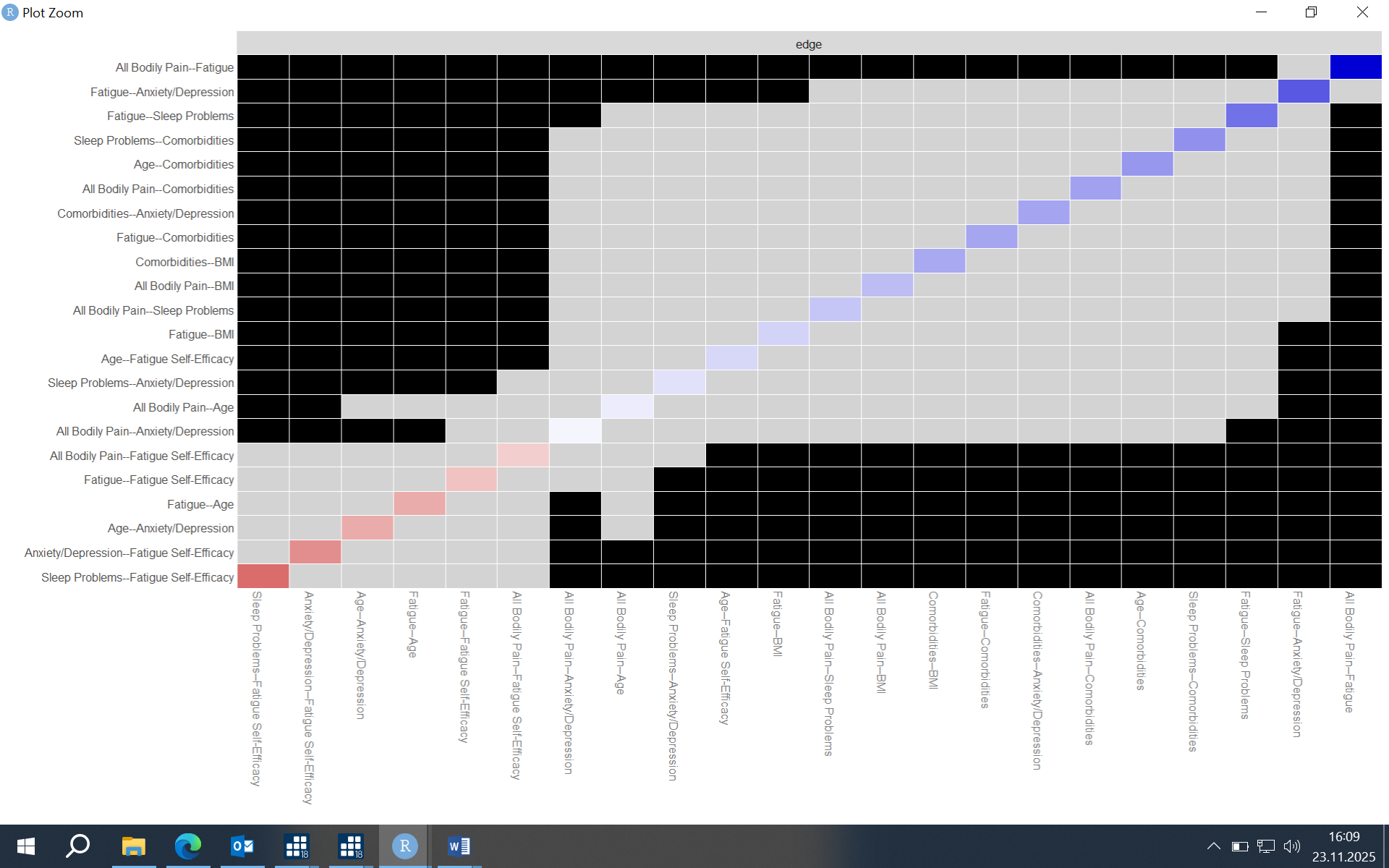


**Supplementary Figure 7:** Sensitivity network of fatigue in people with hand OA (N=300). The green and red colours symbolize conditional positive and negative relationships, respectively, while thicker denser coloured edges represent conditionally higher strength. Numbers represent the weights of these regularized and partial correlations between nodes. *Abbreviations*: BMI= Body Mass Index


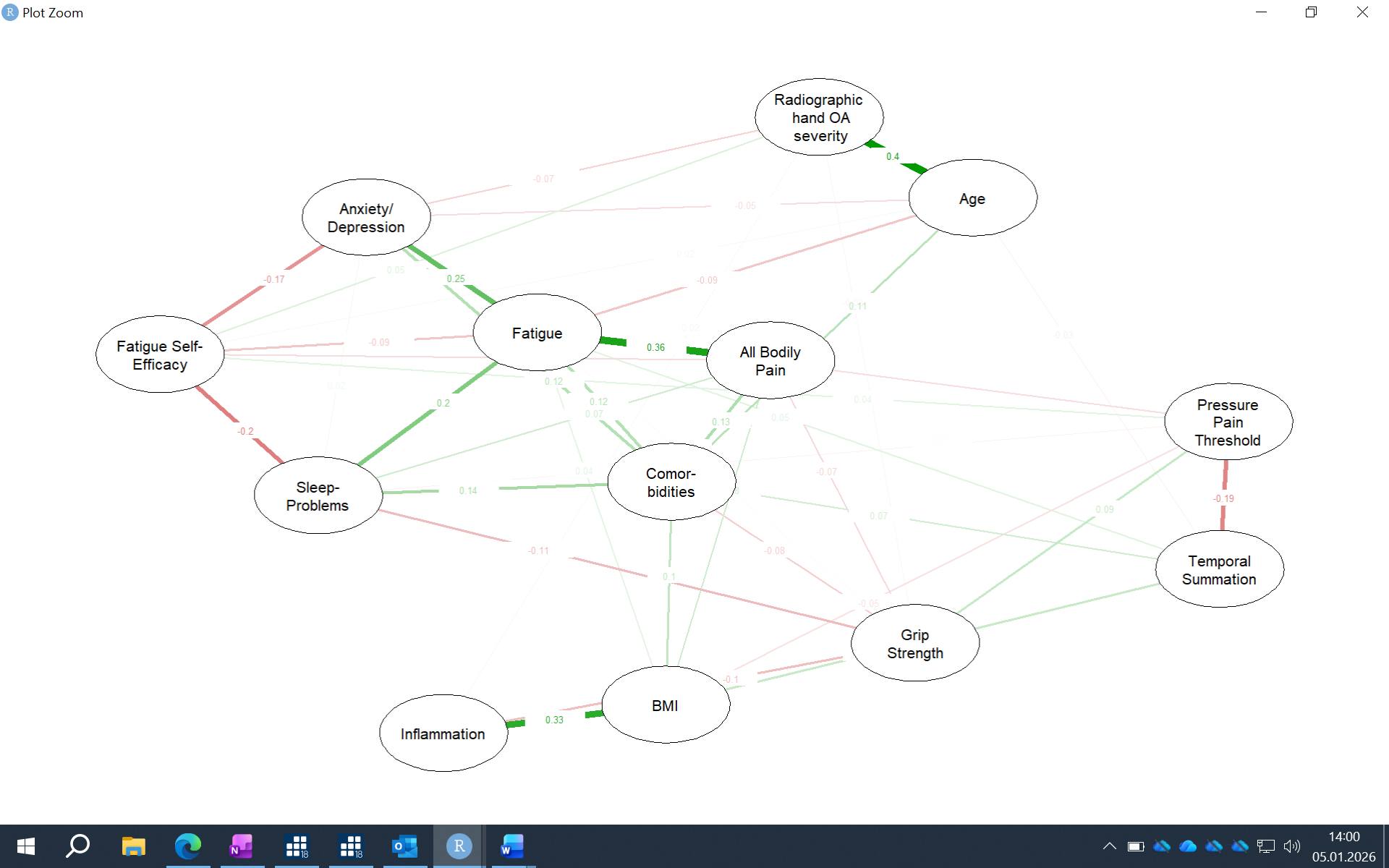


**Supplementary Figure 8:** Centrality indices from the sensitivity network of fatigue presented as relative values (i.e., centrality) in people with hand OA (N=300), with comorbidities, all bodily pain, anxiety/depression and sleep problems showing the highest strength (prioritized measure [6]). *Abbreviations*: BMI= Body Mass Index.


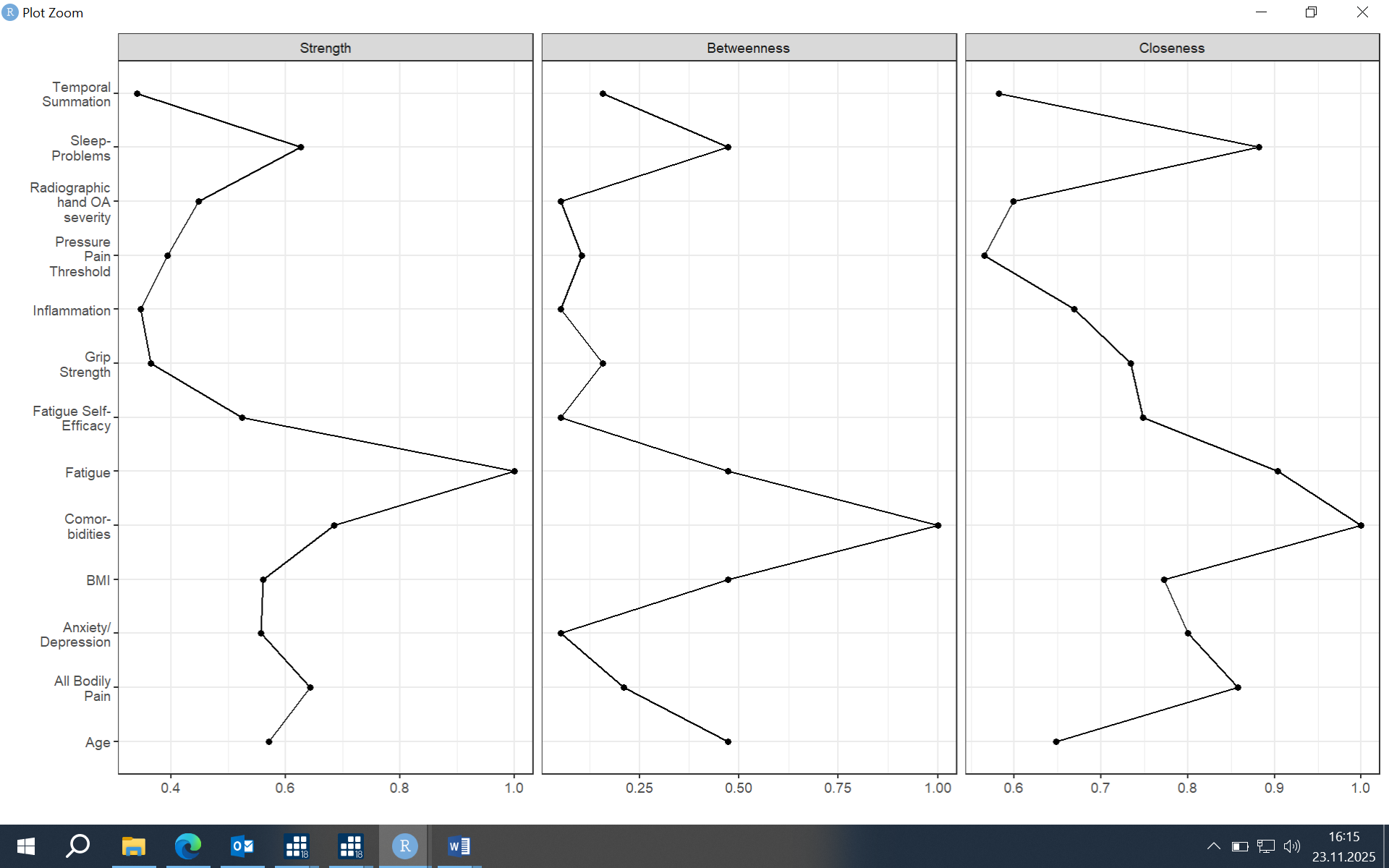


**Supplementary Figure 9:** Test of significant differences across the centrality of each node in the fatigue sensitivity network in people with hand OA (N=300), estimated using bootstrapped values. Higher value represents higher centrality, while black and grey boxes represent *significantly different from* and *non-significantly different* from, respectively. *Abbreviations*: BMI= Body Mass Index


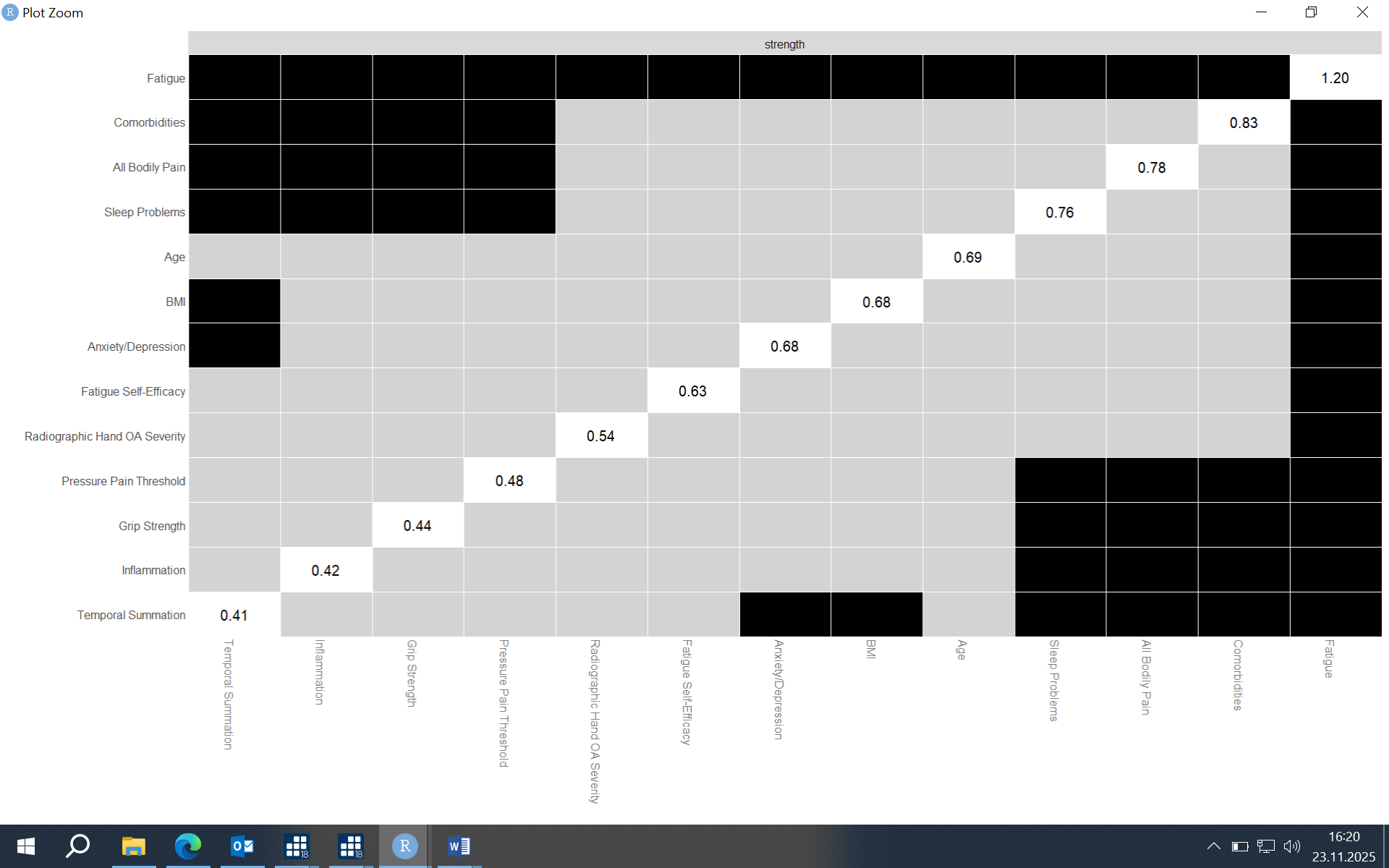


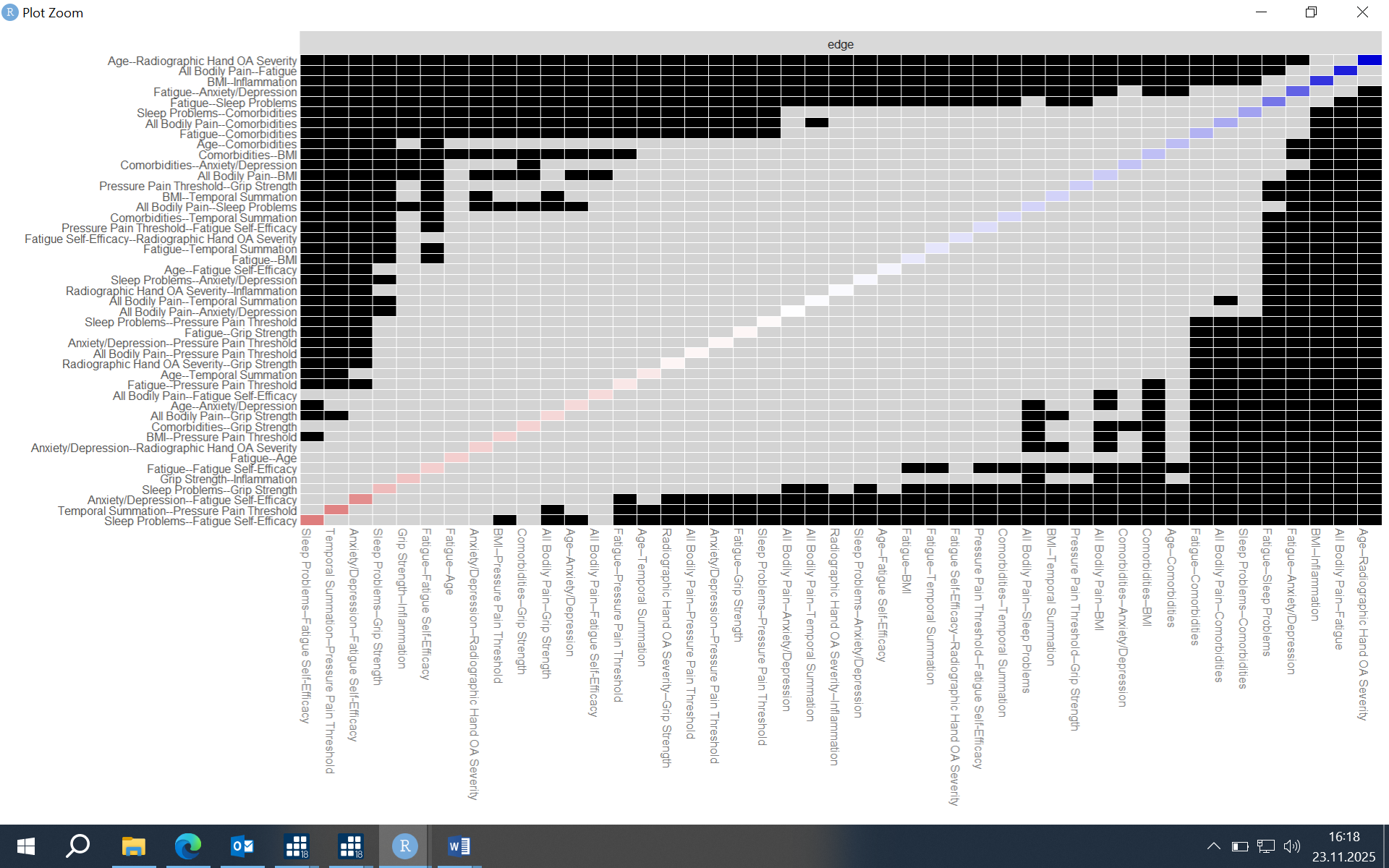
**Supplementary Figure 10:** Test of significantly different edge weights in the *sensitivity* network of fatigue in people with hand OA (N=300), estimated as differences across bootstrapped confidence intervals. Red colours represent negative correlations, while blue colours represent positive correlations. Denser colours represent higher edge weights, while black and grey boxes represent *significantly different from* and *non-significantly different* from, respectively. *Abbreviations*: BMI= Body Mass Index

**Supplementary Table 1**: Presentations of sample characteristics on network variables across the non-imputed versus imputed datasets, including the variability of variance. The fist imputed dataset was used for the analysis.

| **Characteristic** | **Original data** | | **Imputed data** | **SD across imputed datasets**  **N=20** |
| --- | --- | --- | --- | --- |
|  | **Values** | **Missing**  **n (%)** |  |  |
| Age (years), mean (SD) | 60.8 (6.2) | - | 60.8 (6.2) | - |
| NRS fatigue (range: 0-10), mean (SD) | 4.0 (2.9) | 4 (1.3) | 4.1 (2.9) | 0.014 |
| Sleep problems, mean (SD) | 2.3 | 1 (0.3) | 2.3 (1.0) | 0.003 |
| Fatigue self-efficacy (range: 10-100), mean (SD) | 61.5 (22.0) | 3 (1.0) | 61.4 (22.0) | 0.134 |
| HADS (range: 0-42), mean (SD) | 7.3 (6.2) | 9 (3.0) | 7.3 (6.2) | 0.077 |
| Comorbidities (range: 0-45), mean (SD) | 7.7 (4.3) | - | 7.7 (4.3) | - |
| NRS all bodily pain (range: 0-10), mean (SD) | 4.0 (2.3) | 3 (1.0) | 4.0 (2.3) | 0.011 |
| BMI (kg/m^2^), mean (SD) | 26.5 (5.0) | - | 26.5 (5.0) | - |
| KL sum-score (range: 0-128), mean (SD) | 29.9 (19.0) | - | 29.9 (19.0) | - |
| Grip strength (kg, dominant hand), mean (SD) | 21.9 (9.3) | 2 (0.6) | 21.9 (9.3) | 0.026 |
| hs-CRP (raw value), median (IQR)* | 1.6 (0.8-4.3) | 9 (3.0) | 1.6 (0.8-4.3) | 0.013 |
| Temporal summation (range: 0-10), mean (SD) † | 1.6 (1.6) | 2 (0.6) | 1.6 (1.6) | 0.007 |
| Pressure pain threshold (kg/cm^2^), mean (SD) † | 5.5 (2.6) | 9 (3.0) | 5.5 (2.6) | 0.029 |

*Abbreviations*: SD= standard deviation; HADS= Hospital Anxiety and Depression Scale; NRS= Numeric Rating Scale; BMI= Body Mass Index; kg=kilograms; m=meter; KL= Kellgren-Lawrence; hs-CRP= high-sensitive C-Reactive Protein; cm= centimeter; IQR= inter-quartile range

*= log-transformed values were used in the model estimations

†= raw values are presented, but sex standardized were used in analyses.

**Supplementary text 1**: A brief introduction to network analysis

In the current paper, our results were derived by using a relatively new approach to data and for which some readers may be unfamiliar with. Hence, in this text we provide a brief introduction to the methods used, intended to help the reader understand the study results.

The rationale for the analysis is the possibility that factors previously shown to be associated with fatigue may be part of a complex interplay in which several factors may be both potential causes and effects of each other, although no real causal relationship can be derived in a cross-sectional analysis. Nevertheless, network models can showcase and discover such mutual and complex structures by estimating conditional and regularized weighted partial correlations (edges) between the factors of interest (nodes) which can be presented graphically. Hence, network models can answer different research questions compared to e.g., linear regression, in which a directed causal pathway is assumed to exist. Partial correlation refers to the estimation of a direct relationship between every pair of variables, controlling for all other variables in the model. However, to derive an interpretable network, weak or spurious relationships can be shrunk to zero (and thereby not displayed) using the ‘least absolute shrinkage and selection operator’ (LASSO). LASSO is a regularization technique to reduce model complexity or density, which, combined with a tuning parameter selected by the researcher, effectively provides the best model fit as defined by the lowest Extended Bayesian Criterion (EBIC). Hence, the researcher can define “weak” by selecting a tuning parameter aligning with the purpose of the analysis, balancing exploration and confirmation. Thus, the presence of an edge means that the partial correlation observed is strong enough to be included in the model. In the current analysis, as cited in the paper, a tuning parameter recommended by Epskamp et al (2018) was used [6]. Because weak correlations are not part of the final model, LASSO enables researchers to estimate reliable networks even with smaller sample sizes as covariate structure in the data are based on relatively few edges (parameters).

Although networks are often estimated, Epskamp et al propose that the accuracy of networks has received less attention. The use of bootstrapping to define and compare confidence interval overlaps between edges is presented and recommended to solve these problems [6]. However, by shrinking the data (LASSO), the variability of edge-weights cannot be estimated without bias if parametric bootstrapping methods are used to define confidence intervals (CIs). However, a non-parametric alternative (including replacement of observations) is available, and recommended for this purpose, especially for assessment of an edge’s CI width and its overlap with CIs from other edges. A difference test also exists to assess the extent to which the accuracies of two edges differ, estimated by calculating the difference of the bootstrapped weights followed by constructing a CI for the differences, allowing for a null hypothesis to identify the answer of interest (i.e., by assessing if zero is part of the new CI). In this context, adjusting for multiple testing is not feasible [6].

A node is regarded as central if its *strength*, i.e., the sum of the weights of edges connected to that node, independent of direction of the associations included, is high. A *relative* centrality is the normalized value of each node’s centrality, showing a node’s strength relative to the other nodes, making interpretability of hierarchical patterns easier to understand (higher values indicate more “local influence”). While centrality may also include evaluations of betweenness (how often a variable (node) acts as a bridge between others) and closeness (evaluation of path distances, a node may “reach” other nodes quickly), these are regarded as less reliable than strength. However, methods exist to test the stability of the hierarchical stability of node centrality. To do so, Epskamp et al recommend re-estimate the centrality from smaller fractions of the sample (by case-dropping), which can be done by bootstrapping as well. In this context, a correlation stability coefficient can be estimated (CS), with a value of “*0.7 representing the maximum proportion of cases that can be dropped, such that with 95 % probability the correlation between original centrality indices and centrality of networks based on subsets is 0.7 or higher*” [6]. Ideally, this proportion should be higher than 50% of the sample.

Further reading may also include Masuda et al (2025) ***Introduction to correlation networks: Interdisciplinary approaches beyond thresholding,*** and Hevey (2018) **Network analysis: a brief overview and tutorial.**
